# Editorial: New trends in natural product research for inflammatory and infectious diseases

**DOI:** 10.3389/fphar.2022.975079

**Published:** 2022-08-15

**Authors:** Jaime Ribeiro-Filho, Yanna Carolina Ferreira Teles, John Ogbaji Igoli, Raffaele Capasso

**Affiliations:** ^1^ Oswaldo Cruz Foundation (FIOCRUZ), Eusébio, CE, Brazil; ^2^ Department of Chemistry and Physics, Federal University of Paraíba, João Pessoa, PB, Brazil; ^3^ Department of Chemical Sciences, Pen Resource University, Gombe, Nigeria; ^4^ Department of Agricultural Sciences, University of Naples Federico II, Portici, Naples, Italy

**Keywords:** natural products, inflammation, infection, drug development, preclinical research, pharmacology

## Introduction

Inflammatory and infectious diseases have a high prevalence, morbidity, and mortality rates worldwide (Vos et al., 2020). Despite the availability of a considerable number of drugs for the management of these diseases, there are several conditions in which the drugs are ineffective or have side effects that limit their use (Miranda et al., 2021).

Two major challenges with respect to infectious diseases are the presence of a few therapeutic options for the treatment of neglected diseases and the rapid emergence of multi-drug resistant organisms, which has limited the effectiveness of virtually all classes of antimicrobials (Fisher et al., 2018; Murray et al., 2022). Hence there is an urgent demand for the development of novel, safer, and more effective drugs and pharmaceutical formulations (Blasco et al., 2017).

Natural products, especially medicinal plant-derived secondary metabolites, represent an important source of new chemical entities that can be used in pharmacological research especially for inflammatory and infectious diseases. While the present technology, industry, and pharmaceutical market scenario have resulted in decreased patronage of natural products in drug development in the last decades, it is hoped that the development of new technologies will improve drug discovery from natural products and therefore natural products research remains an important field of scientific investigation (Li and Vederas, 2009).

The present Research Topic is intended to collate manuscripts reporting or describing active pharmacological principles of extracts presenting well characterized and quantified natural products with effectiveness against inflammatory and infectious diseases. A total of 17 manuscripts were published reporting anti-inflammatory, antiviral, antibacterial and antiparasitic activities.

## Anti-inflammatory activity

Most of the studies reported in our RT demonstrate the role of phytochemicals and herbal formulations in experimental models of inflammation. A review by Oliveira-Costa and others (Oliveira-Costa et al.) discussed the ant-inflammatory activities of betulinic acid, a lupane-type pentacyclic triterpene that is commonly isolated from *Betula* species. Betulinic acid was found to modulate the production of key inflammatory mediators *in vitro* and *in vivo*, in different models of inflammation, which is probably due to the inhibition of nuclear factor kappa-B (NF-κB) and mitogen-activated protein kinase (MAPK) pathways. The compound has served as a prototype for a large number of derivatives with significant potential in drug development such as 3-Deoxy-3β-((6-(2-heptanoyl-3-oxocyclopent-1-en-1-yl)amino) hexanamido) betulinic acid. Another review by the same group (Meira et al.) highlighted the therapeutic applications of physalins (a class of compounds commonly found in the Solanaceae family) in anticancer, immunomodulatory, and antiparasitic activities. Physalin B and F had the most potent pharmacological effects, but their mechanism of action and toxic properties remain to be described. Kuang et al. investigated the anti-inflammatory activity of the sesterterpenoid fusaproliferin and its analogues in RAW264.7 macrophages and zebrafish embryos stimulated with lipopolysaccharide (LPS). The activity of the sesterterpenoid was associated with the inhibition of nitric oxide (NO), reactive oxygen species (ROS), and cytokine production, as well as with a decreased expression of inflammatory enzymes such as nitric oxide synthase (iNOS) and cyclooxygenase-2 (COX-2). As for betulinic acid, the mechanism of action of fusaproliferin and its analogues was shown to involve the inhibition of the TLR4-mediated activation of NF-kB and MAPK signaling pathways. In fact, NF-κB is a family of transcription factors that play crucial roles in cell activation, proliferation, and survival, and therefore is a key molecular target in anti-inflammatory research (Liu et al., 2017).

Four manuscripts investigated the effects of natural products on the NLRP3 inflammasome, an intracellular multiprotein complex with significant roles in inflammation and host defense. Piperlongumine, an alkaloid isolated from *Piper longum* L. was identified as an NLRP3 inhibitor that acts by interrupting the assembly of the inflammasome. In addition, the significant *in vivo* activity demonstrated in LPS-induced endotoxemia and MSU-induced peritonitis, indicated the potential application of the alkaloid in NLRP3-associated diseases (Shi et al.). (-)-Epicatechin, a flavonoid known for antioxidant and anti-inflammatory activities, was found to significantly inhibit the inflammatory response in MSU-induced acute gouty arthritis. The compound caused significant inhibition of inflammatory mediator production, edema development, and leukocyte infiltration through inhibition of NLRP3 Inflammasome and the NF-κB signaling pathway (Wu et al.). *Qingwenzhike* (QWZK), a Traditional Chinese medicine preparation derived from recombination of ancient Chinese classical prescriptions, was chemically characterized by linear ion trap/electrostatic field orbital trap tandem high-resolution mass spectrometry (UHPLC-LTQ-Orbitrap MS) and investigated for its protective effects on LPS-induced acute lung injury (ALI) model induced in rats. A total of 99 compounds (mostly flavonoids) were identified in QWZK, which demonstrated protective effects on LPS-induced ALI, possibly by inhibiting TLR4/NF-kB signaling pathway and NLRP3 inflammasome activation (Zhang et al.). Together, these findings point to NF-kB and NLRP3 as potential targets in natural product-based anti-inflammatory drug development. Similar mechanisms were demonstrated in a study by Lu et al. with the species *Tetrastigma hemsleyanum* Diels et Gilg, (Vitaceae), a Chinese medicinal herb (popularly known as *Sanyeqing*) that is traditionally being used to treat inflammation. The authors demonstrated that polysaccharides obtained from this plant ameliorated LPS-induced acute respiratory distress syndrome in mice through modulation of TLR2/TLR4-NF-κB, NLRP3/caspase and JAK/STAT signaling pathways, stimulating further research on the benefits of these molecules on respiratory disorders such as the coronavirus disease (COVID-19). An article by Yan et al. reported the use of a murine model of colitis to demonstrate the therapeutic effects of *Guchangzhixie* capsule, an established drug in the Chinese Pharmacopoeia 2020. It is composed of *Mume fructus, Zingiberis rhizoma, Aucklandiae radix, Corydalis rhizome, Coptidis rhizoma,* and *Papaveris pericarpium*. It was observed that the anti-inflammatory properties of *Guchangzhixie* result from modulation of macrophage polarization and inflammatory mediator production, favoring mucosal healing. These latter studies demonstrate the relevance of traditional Chinese medicine in anti-inflammatory drug research.

## Antiviral activity

The current pandemic of COVID-19 led many researchers working on drug discovery to search for novel sources and compounds that could be useful as antivirals or as adjuvants on the treatment of COVID-19 symptoms or complications. Although the vaccines are available and effective to avoid severe COVID-19, the threat of SARS-CoV-2 new variants makes the development of antiviral therapies an urgent need. Several studies were reported investigating natural products with potential action against SARS-CoV-2. Singla et al., carried out a wide review on the role of intestinal microbiota and pro-inflammatory markers on COVID-19. Additionally, they reviewed natural products that could combat the SARS-CoV-2 virus. The authors described 70 phytochemicals and ten polyherbal formulations which were scientifically analyzed against the SARS-CoV-2 virus, showing the great potential of bioresources on prevention and treatment of COVID-19 complications (Chao et al.).

In another research, Kolev et al. investigated the antiviral effects of *Echinacea purpurea* focusing on SARS-CoV-2 virus. The plant species was previously shown to possess antiviral and immuno-modulating properties, indicating that it could be useful against SARS-CoV-2. In an exploratory clinical study with 120 volunteers, it was demonstrated that the use of a pharmaceutical preparation of *Echinacea purpurea* extract potently reduced SARS-CoV-2 infections and viral loads as part of an overall effect on viral respiratory tract infections (Kolev et al.), suggesting that this preparation could be complementary to other activities, such as vaccination and use of face masks, that could attenuate the development of severe COVID-19. Using a different approach, Yeh et al. developed a computational method to select herbs from Traditional Chinese Medicine (TCM) with the greater potentials to be active against SARS-CoV-2 binding and replication. Using current procedures, the authors established novel *in silico* methods to construct a comprehensive map of TCM drugs that possess potential for SARS-CoV-2 prevention and treatment. According to the *in silico* predictions, Honeysuckle (*Lonicera japonica*) and Huangqi (*Astragalus membranaceus*) were shown to have therapeutic potential by blocking the binding of spike protein-ACE2, suppressing SARS-CoV-2 replication and the inflammatory phase by targeting cytokines. The preliminary results were validated by collecting the selected herbs and evaluating their anti-viral activity *in vitro*. Based on their findings, the authors demonstrated that TCM candidates could be prioritized through *in silico* predictions, followed by validation using various anti-viral activity assays (Yeh et al.).

Regarding other viral infections, Chao et al. studied the Chinese herb *Andrographis paniculate* (*Chuanxinlian*). They identified twelve compounds produced by the plant and evaluated their activity against enterovirus 71 (EV71), one of the most important enteroviruses that cause hand, foot, and mouth disease accompanied by neurological complications. In their study they demonstrated that bioactive compounds of *Chuanxinlian* act either by protecting EV71-infected RD cells from sub-G1 arrest or possessing IFNγ-inducer activity, thus it may be feasible to develop anti-EV71 agents.

The relevance of Traditional Chinese Medicine in the treatment of all kinds of diseases was reinforced by a Chinese research group working on hepatitis B virus (HBV). They investigated the anti-HBV effect and the related mechanisms of action of a Chinese patent medicine *Liuweiwuling*. In spite of the fact that *Liuweiwuling* tablets are licensed in Chinese patent medicine with indications as anti-inflammatory and to be used by patients with chronic HBV infection, its anti HBV effect remained unclear. In the report Ge et al. demonstrated the potent inhibitory effect on both wild-type and entecavir-resistant HBV, which might be associated with increasing IFN-β and IFN-γ production (Ge et al.).

## Antibacterial and antifungal activities

The interest in novel antimicrobial compounds has significantly increased in the last years due to the lack of effectiveness of conventional drugs against resistant microorganisms such as bacteria and fungi. In this context, research by Singh et al. evaluated the effects of a bacteriophage cocktail obtained from different water sources on septicemia caused by colistin-resistant *Klebsiella pneumoniae* in mice. The authors observed that while the lower dose (1 × 10^5^ PFU/mouse) had a protective effect, the higher dose (1 × 10^12^ PFU/mice) was fatal in the early stages of septicemia but not at the later stages. Moreover, the outcome observed in the high-dose phage-treated mice were associated with elevated IL-6 concentrations. This was the report on the biological role of a natural product that was not derived from a plant. It is a stimulus to the investigation of other natural sources in anti-inflammatory and antimicrobial drug development.

As some diseases are caused by bacterial and fungal co-infections, compounds with a wide spectrum of action may represent promising alternatives as antimicrobial agents. In this context, thymol a widely studied terpene, had its anti-infective potential investigated in a model of caries caused by *Candida albicans* and *Streptococcus mutans* co-infection. The study carried out by Priya et al. demonstrated that the monoterpene inhibited the growth of both pathogens, in addition to inhibiting several virulence factors of these microorganisms *in vitro*. Moreover, *in vivo* studies using a *Galleria mellonella* model indicated significant inhibition of infection under a single and dual state in the absence of significant toxicity, supporting the application of thymol in the development of pharmaceutical formulations for the treatment of caries.

## Antiparasitic activity

Parasitic diseases are a great threat to billions of people especially in the tropical regions of the globe where there is prevalence of neglected diseases, such as filariasis, schistosomiasis and leishmaniases (Igoli et al., 2022). In this category, the first manuscript reported on the *in vitro* activity of BA5 (a betulinic acid derivative) against different species of *Leishmania* as well as its mechanism of action. The authors reported that BA5 inhibited the proliferation of promastigote forms of *Leishmania amazonensis*, *Leishmania major*, *Leishmania braziliensis* and *Leishmania infantum.* Using electron microscopy and flow cytometry, it was demonstrated that promastigotes incubated with BA5 presented membrane blebbing, flagella damage, increased size, body deformation, and that parasite mortality is mainly caused by apoptosis-like death and arrested cell cycle in G0/G1 phase (Magalhães et al.). In the second manuscript, Okoh et al. used cheminformatics to investigate two natural compounds and their potential for use against Malaria. Molecular dynamics was used to compare the binding affinity of artesunate and azadirachtin to the active site of Gephyrin E, a multi-domain scaffolding protein of inhibitory post-synapses. The results provided evidence that artesunate has comparatively better binding affinity to Gephyrin E than azadirachtin, although they presented evidence that azadirachtin may be more effective as an anti-malarial agent than artesunate.

## Conclusion

The Research Topic ‘New trends in natural product research for inflammatory and infectious diseases’ was effective in bringing together worthy studies and contributions on the subject of inflammatory and infectious diseases, highlighting the relevance of natural products in current pharmacological research in a pre-clinical context.
